# Vitamin D_3_ enhances the response to cisplatin in bladder cancer through VDR and TAp73 signaling crosstalk

**DOI:** 10.1002/cam4.2119

**Published:** 2019-04-10

**Authors:** Brittany L. Bunch, Yingyu Ma, Kristopher Attwood, Lauren Amable, Wei Luo, Carl Morrison, Khurshid A. Guru, Anna Woloszynska‐Read, Pamela A. Hershberger, Donald L. Trump, Candace S. Johnson

**Affiliations:** ^1^ Department of Pharmacology and Therapeutics Roswell Park Cancer Institute Buffalo New York; ^2^ Department of Biostatistics and Bioinformatics Roswell Park Cancer Institute Buffalo New York; ^3^ National Institute on Minority Health and Health Disparities National Institutes of Health Bethesda Maryland; ^4^ Department of Pathology Roswell Park Cancer Institute Buffalo New York; ^5^ Department of Urology Roswell Park Cancer Institute Buffalo New York; ^6^ Inova Schar Cancer Institute Falls Church Virginia

**Keywords:** bladder cancer, cisplatin, TAp73, VDR, vitamin D_3_

## Abstract

**Background:**

Vitamin D_3_ (VitD) deficiency is linked to increased incidence and worse survival in bladder cancer (BCa). In addition to cystectomy, patients are treated with cisplatin‐based chemotherapy, however 30%‐50% of patients do not benefit from this treatment. The effects of VitD deficiency on response to chemotherapy remain unknown.

**Methods:**

To test effects of VitD supplementation on the response to cisplatin we analyzed patient serum VitD levels and correlated that with survival. In vivo, VitD deficient mice were treated with cisplatin, with or without pretreatment with the active VitD metabolite, 1,25 dihydroxyvitamin D_3_ (1,25D_3_). Lastly, using BCa cell lines, T24 and RT‐112, the mechanism of action of 1,25D_3_ and cisplatin combination treatment was determined by apoptosis assays, as well as western blot and RT‐PCR.

**Results:**

In this study, we determined that low serum 25 hydroxyvitamin D_3_ (25D_3_) levels was significantly associated with worse response to cisplatin. Pretreating deficient mice with 1,25D_3_, reduced tumor volume compared to cisplatin monotherapy. In vitro, 1,25D_3_ pretreatment increased the apoptotic response to cisplatin. 1,25D_3_ pretreatment increased expression of TAp73 and its pro‐apoptotic targets, in a VDR dependent manner. VDR and its transcriptional targets were induced after 1,25D_3_ treatment and further increased after the combination of 1,25D_3_ and cisplatin in a TAp73 dependent manner.

**Conclusions:**

Our data suggest that VitD deficiency could be a biomarker for poor response to cisplatin, and pretreating with VitD can increase the apoptotic response to cisplatin through VDR and TAp73 signaling crosstalk.

## 1 INTRODUCTION

Bladder cancer (BCa) is the seventh most common cancer in the US, with 81 000 new cases and 17 000 deaths in 2018.^1^ Muscle invasive bladder cancer (MIBC) has worse survival, is more likely to become metastatic, and is treated with multi‐agent cisplatin‐based neoadjuvant chemotherapy and radical cystectomy.^2^ Two major limitations of neoadjuvant chemotherapy are delaying cystectomy in patients with poor response to cisplatin (~30%‐50%), and the lack of available biomarkers to identify patients unlikely to respond to cisplatin.^3^ Two years after cystectomy 50% of patients relapse with metastatic disease, suggesting a high degree of initial micro‐metastasis.^4^ Metastatic MIBC has poor survival (5‐year survival ~15%) and is treated by systemic chemotherapy.^2^, ^5^ Improving the effectiveness of cisplatin could improve overall survival.

Vitamin D_3_ (VitD) deficiency is linked to increased incidence and worse survival in a variety of tumors, including bladder, prostate, breast, and colorectal.^6^, ^7^, ^8^, ^9^ Low serum 25 hydroxyvitamin D_3_ (25D_3_), the circulating VitD metabolite, is associated with an increased incidence and worse survival in BCa.^7^, ^8^,25 dihydroxyvitamin D_3_ (1,25D_3_), the active VitD metabolite, has antitumor properties in many cancers and has been shown to increase the response to Bacillus Calmette‐Guerin (BCG) in non‐MIBC.^10^, ^11^, ^12^, ^13^ Whether VitD status affects a patient's response to chemotherapy is unknown. If VitD deficiency affects a patient's response to cisplatin, 1,25D_3_ may have therapeutic potential in MIBC.

p53 is mutated in ~50%‐60% of MIBC cases.^14^ In a p53‐independent mechanism, p73 is activated after cisplatin‐induced DNA damage. p73, a p53 family member rarely mutated in cancer, has both multiple isoforms.^15^ The anti‐apoptotic isoform, ΔNp73, is transcribed from an intrinsic promoter site and is transcriptionally inactive.^16^, ^17^ TAp73, the full length pro‐apoptotic isoform, transcribes similar targets as p53 such as BAX and NOXA.^18^, ^19^ The ratio of TA/ΔN isoforms determines the response to chemotherapy.^20^, ^21^, ^22^ Clinical response to cisplatin in BCa does not correlate with p53 mutation status.^14^, ^23^ In addition, cisplatin‐resistant BCa cell lines lose the ability to induce p73.^24^ These findings suggest an important role of p73 in the apoptotic response to cisplatin.

1,25D_3_ increases the antitumor response to cisplatin in a variety of preclinical settings.^25^, ^26^ However, the importance of VitD sufficiency in the response to cisplatin has not been investigated in BCa. In the current study, we investigated how VitD deficiency altered response to cisplatin in vivo and in patients. We then investigated the mechanism of action of 1,25D_3_ and cisplatin combination therapy. Our data provides rationale to determine the serum 25D_3_ level in patients with MIBC prior to receiving neoadjuvant chemotherapy and consider VitD supplementation as a pretreatment strategy.

## 2 METHODS

### 2.1 Cell lines

Human BCa cell lines T24, RT‐112, and 253J were purchases from ATCC (Manassas, VA). T24 cells were cultured in McCoys 5A media with l‐glutamine supplemented with 10% FBS and 1% penicillin/streptomycin sulfate and used within 6 months of purchase. RT‐112 and 253J cells were cultured in RPMI media with L‐glutamine supplemented with 10% FBS and 1% penicillin/streptomycin sulfate. All cells were tested for mycoplasma within the past 4 years (PCR Mycoplasma Detection Kit, MD Bioproducts). Cell lines were not authenticated after purchase.

### 2.2 Tissue microarray

The BCa tissue microarray at Roswell Park was stained with anti‐VDR. Nuclear protein expression was digitally scanned using Aperio Scanscope (Aperio Technologies, Inc., Vista, CA) with 20× bright‐field microscopy. VDR expression was summarized by patient characteristics using the mean and standard deviation, and the median and IQR (25th‐75th percentiles). The association between patient characteristics and expression were evaluated using parametric *t*‐tests or one‐way ANOVA; and the non‐parametric Mann‐Whitney U or Kruskal‐Wallis tests. The survival outcomes (overall and disease‐specific) were summarized by expression level using standard Kaplan‐Meier methods. Estimates of the median time and 3/5‐year rates were obtained with 95% confidence intervals. Comparisons were made using the log‐rank test.

### 2.3 In vivo deficiency vs sufficiency

In vivo protocols were approved by the Institutional Animal Care and Use Committee at Roswell Park Cancer Institute and performed in compliance with institutional guidelines and regulations. Ten female nude mice, 4 weeks old, were placed on a VitD deficient diet (Research Diets, 25 IU) and ten female nude mice, 4 weeks old, were placed on a VitD sufficient diet (Research Diets, 1000 IU) for the course of the experiment. After 6 weeks on the diets, mice were inoculated with 3 × 10^6^ T24 cells in Matrigel:HBSS (10:1) in the right flank. When tumors reached approximately 300 mm^3^, mice were randomized (n = 5 per group) and given IP injections of 5 mg kg^−1^ of c (100 μL volume) or saline once a week for 3 weeks. Tumor volume was measured with calipers (calculated by (length × width^2^)/2) and mouse weights were recorded every other day. Fractional tumor volume was calculated using the start of treatment as day 0. Serum 25D_3_ levels were analyzed by Heartland Assays using a radioimmunoassay.

### 2.4 In vivo treatment with 1,25D_3_ and cisplatin

Forty female nude mice, 4 weeks old, were placed on the VitD deficient diet for 6 weeks. Mice were inoculated with T24 tumors as described. When tumors reached approximately 200 mm^3^, mice were randomized and treated with either saline, 1,25D_3,_ cDDP, or 1,25D_3_ and cDDP (n = 10 per group). 0.625 μg of 1,25D_3_ was given IP on Monday, Wednesday, and Friday, and 5 mg kg^−1^ of cDDP was given on Friday (100 μL volume). Serum and kidneys were harvested from 3 mice per treatment group 8 hours after the first week of treatment. Tumor volume was measured with calipers (calculated by (length × width^2^)/2) and weights were recorded. Fractional tumor volume was calculated using the start of treatment as day 0. An *F*‐test about the treatment‐time interaction was used to evaluate differences in tumor growth rates between treatment groups. Post‐hoc comparisons were made using Holm‐Bonferroni adjusted *F*‐tests about the appropriate linear contrast of model estimates. Serum metabolites of VitD were analyzed by Heartland Assays using a radioiummunoassay.

### 2.5 Patient serum analysis and survival

BCa patient serum samples were obtained from Roswell Park Cancer Institute Data Bank and Biorepository. Serum 25D_3_ levels were analyzed by Heartland Assays using LC/MS/MS. Patients were grouped as low (25D_3_ <20 ng mL^−1^) or high (25D_3_ ≥20 ng mL^−1^). Survival outcomes were summarized using Kaplan‐Meier methods: the 3/5 year and median survival were obtained with 95% confidence intervals, comparisons made using the log‐rank test.

### 2.6 MTT assay

Cells were pretreated with EtOH or increasing concentrations of 1,25D_3_ (10 nmol L^−1^‐1 μmol L^−1^) for 24 hours and followed by increasing concentrations of cDDP (0.1 μg mL^−1^‐10 μg mL^−1^) for 48 hours. Cell growth was assessed by MTT assay as previously described.^27^ Fraction affected was calculated using the equation (1‐OD value of treatment cells/OD value of control ethanol treated cells). Combination indexes (CI) were determined using CalcuSyn software. A CI <1 denotes synergy.

### 2.7 Clonogenic assay

Cells were pretreated with EtOH or 100 nmol L^−1^ 1,25D_3_ for 24 hours and then treated with 0.1 μg mL^−1^ cDDP for 48 hours or left untreated. Cells were trypsinized using 0.25% trypsin, counted and replated in triplicate at a density of 150 cells per well in a 6‐well plate. Colonies formed over 10 days and were fixed and stained with 40% methanol crystal violet.

### 2.8 shRNA lentiviral transduction

Plasmid DNA was prepared by adding 10 μL of each glycerol stock to 5 mL of 2A‐LB broth (low salt) and 100 μg mL^−1^ of carbenicillin. DNA was isolated using a DNA mini prep kit (Zymo Research). shRNA lentivirus was produced in HEK293T cells using pCMV‐dR8.74, VSV‐G, and Lipofectamine 2000. T24 and RT‐112 cells were transduced with the shRNA lentivirus containing media and 4 μg mL^−1^ of polybrene. Cells were selected with puromycin (2.5 μg mL^−1^). shRNA knockdown was confirmed at both mRNA and protein levels using qRT‐PCR and Western blot analysis. TAp73 shRNA sequence: TGCTGTTGACAGTGAGCGAGGCCATGCCTGTTTACAAGAATAGT GAAGCCACAGATGTATTCTTGTAAACAGGCATGGCCCTGCCTACTGCCTCGGA.

### 2.9 siRNA transfection

Cells were plated in antibiotic‐free complete media. 5 μmol L^−1^ siRNA stock solutions were prepared in 1× siRNA buffer. siRNA was diluted in serum‐free media. DharmaFECT transfection reagent 1 was diluted in serum‐free media. siRNA and DharmaFECT were mixed and incubated for 20 minutes at room temperature. Antibiotic‐free complete media was added to a final siRNA concentration of 25 nmol L^−1^. Media was removed from cultured cells and replaced with siRNA containing media and incubated at 37°C in 5% CO_2_ for 24 hours. siRNA knockdown was confirmed at both mRNA and protein levels using qRT‐PCR and Western blot analysis.

### 2.10 Real‐time quantitative RT‐PCR

RNA was isolated using Directzol RNA MiniPrep (ZymoResearch) and quantified. cDNA was made using Transcriptor First Strand cDNA Synthesis Kit (Roche) and 1 μg of total RNA. qRT‐PCR was performed on an Applied Biosystems 7300 real‐time system (Applied Biosystems, Foster City, CA) using iTaq^™^ Universal SYBR^®^ Green Supermix (Bio Rad), 500 nmol L^−1^ of forward and reverse primers, dH_2_O, and 1 ul of cDNA. Standard thermal cycler conditions were used. Primer pairs were purchases from IDT, sequences can be found in Supplementary Methods.

### 2.11 Western blot analysis

Cell lysates were prepared using Triton X‐100/SDS lysis buffer supplemented with 4% protease inhibitor cocktail and 1% phosphatase inhibitor cocktail. Protein concentrations were determined using Bio‐Rad Protein Assay Dye Reagent and made into samples with 30 μg of protein. Samples were run on a 4%‐20% SDS‐PAGE gel and transferred using a Bio‐Rad Semi‐dry Transfer Cell. Membranes were blocked with 5% milk and probed with antibodies described in Materials.

### 2.12 Statistical analysis

All experiments were performed in biological and technical triplicate and graphically represented as the average ± SE. ANOVA followed by post‐hoc Bonferroni comparisons were used to determine significance between treatment groups unless stated otherwise. GraphPad Prism was used to calculate significance (*P* value <0.05). Asterisks alone denote significance compared with control treated samples, asterisks and lines denote significance between treatment groups.

## 3 RESULTS

### 3.1 High VDR expression is associated with improved survival

**Figure 1:**
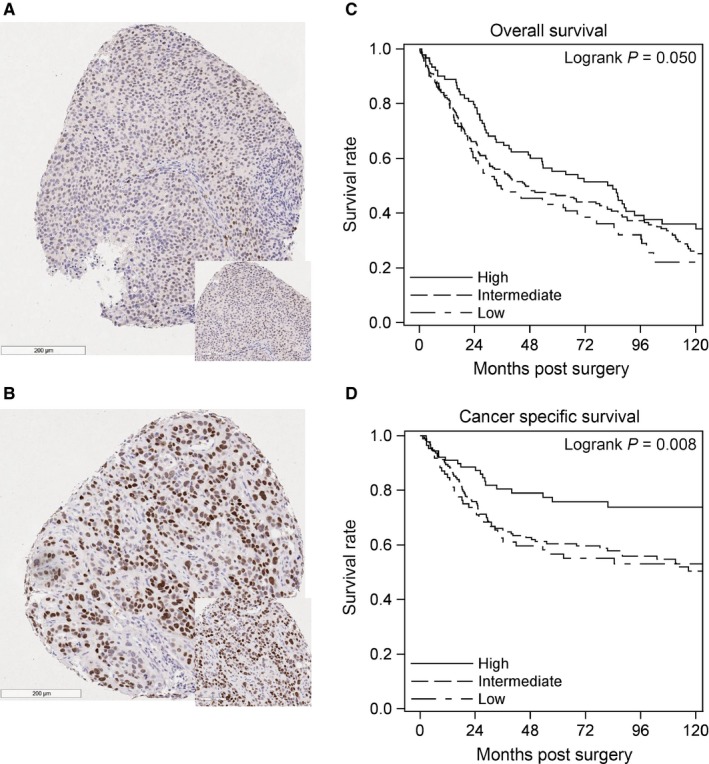
High VDR expression is associated with improved survival in BCa patients. A patient derived BCa TMA containing 359 samples was stained with anti‐VDR. Nuclear expression was quantified as an H‐score based on % positive nuclei and staining intensity. Representative images of a (A) low and (B) high H‐score are shown. (C) Overall survival and (D) disease‐specific survival were summarized by expression level (quartiles, High, Intermediate, and Low) using standard Kaplan Meier methods. Overall and disease‐specific survival increased in patients with high (>Q3, H‐score > 140.60) VDR expression compared with those with intermediate or low expression (*P* = 0.05, 0.008 respectively). Comparisons were made using the log‐rank test

To evaluate the clinical significance of VDR protein expression in BCa tumor tissues, 359 primary tumor samples collected at Roswell Park Cancer Institute compiled on tissue microarrays (TMAs) were stained with anti‐VDR. Patient demographics and clinical characteristics are listed in Tables S1 and S2. H‐score was calculated as a combination of intensity and % positive nuclei. Representative images of low (1.5595) and high H‐scores (232.2660) can be found in Figure 1A‐B. A significant decrease in VDR H‐score was observed in patients with muscle invasive disease compared with superficial disease (Table S3, *P* < 0.001). VDR expression decreased with an increase in grade (*P* = 0.002), clinical T stage, Path AJCC, Path T and Path N stage (Table S3, *P* < 0.001). To study survival, VDR H‐scores were then classified into quartiles (low: H‐score < 45.58, high: H‐score > 140.60, and intermediate: 45.58 ≤ H‐score ≤ 140.60). A significant association was observed with improved overall survival and disease‐specific survival in patients with higher VDR expression (Figure 1C‐D, *P* = 0.05 and *P* = 0.008, respectively).

### 3.2 Vitamin D_3_ sufficient mice have an improved response to cisplatin

**Figure 2:**
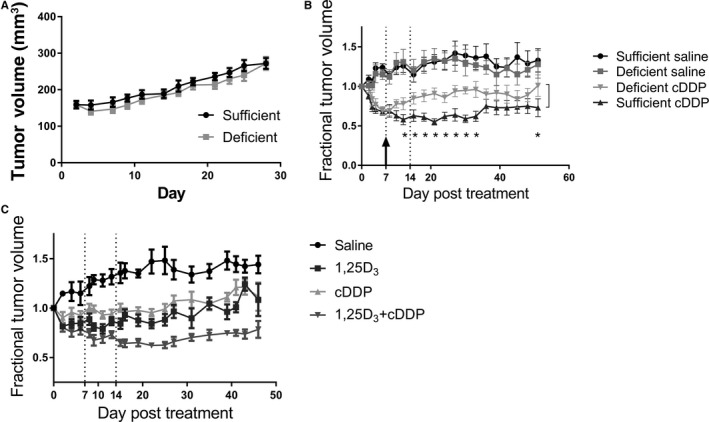
Vitamin D_3_ sufficiency increases the response to cisplatin in vivo, through either dietary intervention or treatment with 1,25D_3_. (A‐B) Twenty female nude mice on a VitD deficient (25 IU) or sufficient (1000 IU) diet were treated with 5 mg kg^−1^ of cisplatin (cDDP) or saline once a week for 3 weeks (n = 5 per group, dotted lines indicated treatment‐days 0, 7, 14). (A) Tumor growth was measured every other day prior to treatment. Tumor growth was not affect by the diets. (B) After treatment, tumor growth was measured every other day and fractional tumor volume was calculated based on tumor volume at the start of treatment (day 0). VitD sufficient mice had a greater reduction in tumor volume and a prolonged response compared with VitD deficient mice (*P < 0.05, comparison between Deficient cDDP and Sufficient cDDP). The arrow represents point in treatment where deficient mice had a maximum response. (C‐D) Forty female nude mice on a VitD deficient diet were treated with saline, 1,25D_3_ (0.625 μg, MWF), cDDP (5 mg kg^−1^, F), or the combination (n = 10 per group). Tumor volume was measured every other day and fractional tumor volume was calculated based on tumor volume at the start of treatment. VitD deficient mice treated with 1,25D_3_ and cisplatin had a significant reduction in tumor growth compared with either monotherapy determined using Holm‐Berferroni adjusted *F*‐test (*P* < 0.001, compared to all treatment groups)

To investigate the importance of serum VitD levels in the response to cisplatin, we placed mice on a VitD deficient (25 IU, serum 25D_3_ 4.16 ± 0.97 ng mL^−1^) or sufficient diet (1000 IU, serum 25D_3_ 17.2 ± 1.12 ng mL^−1^) for 6 weeks to allow serum levels to equilibrate. Mice were inoculated subcutaneously with T24 cells. Tumor growth was not affected by the diets (Figure 2A). Mice were treated with saline or 5 mg kg^−1^ of cisplatin once a week for 3 weeks (Figure 2B, day 0, 7, and 14). Five mg kg^−1^ of cisplatin was determined as the maximum tolerated dose for this schedule (data not shown). After the first dose of cisplatin, all mice had a reduction in tumor volume of ~30% (Figure 2B, black arrow). However, mice on the deficient diet showed no further reduction in tumor volume despite 2 additional treatments and tumor volume returned to baseline. In contrast, mice on the sufficient diet had a maximum reduction of tumor volume of ~50%, which was stably maintained up to 36 days post treatment (*P* < 0.05). Tumor volume in the sufficient mice increased slightly 3 weeks after treatment ended, but maintained a 25% reduction. Cisplatin treatment caused a slight drop in mouse weight (5%‐7%), which was not altered by the different diets (Figure S1A). These data suggest that VitD sufficient mice have an improved response to cisplatin compared with deficient mice.

### 3.3 1,25D_3_ can increase the response to cisplatin in vitamin D_3_ deficient mice

After determining that VitD deficient mice have a worse response to cisplatin, we investigated if pretreating deficient mice with 1,25D_3_ (0.625 μg mouse^−1^, Monday, Wednesday, and Friday (MWF)) could improve response. Mice were placed on a VitD deficient diet (25 IU) for 6 weeks. Mice were inoculated with T24 cells subcutaneously and treated with saline, 1,25D_3_ (0.625 μg mouse^−1^, MWF), cisplatin (5 mg kg^−1^, Friday), or 1,25D_3_ followed by cisplatin. After treatment with 1,25D_3_, serum levels of 1,25D_3_ increased from approximately 93 to >210 pg mL^−1^, the upper limit of the assay. Mice treated with the combination had the largest reduction in tumor volume and a significant reduction in growth rate (*P* < 0.01 compared with all other groups, Figure 2C, Figure S1D). Mouse weights decreased slightly after cisplatin treatment but rebounded prior to the next round of treatment and were not affected by 1,25D_3_ (Figure S1C). These data indicate that pretreating deficient mice with 1,25D_3_ can increase the response to cisplatin in vivo.

### 3.4 High serum 25D_3_ levels associate with improved survival in BCa patients treated with cisplatin

**Figure 3:**
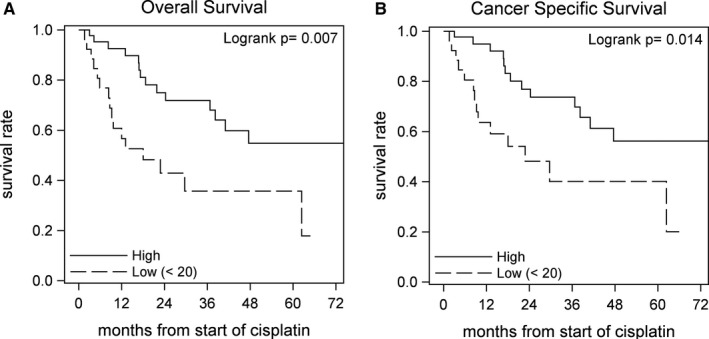
High serum 25D_3_ is associated with improved survival in BCa patients treated with cisplatin. Seventy‐one serum samples from BCa patients treated with cisplatin were analyzed for 25D_3_ using LC/MS/MS. Patients were grouped into high (25D_3_ ≥ 20 ng mL^−1^) or low (25D_3_ < 20 ng mL^−1^) groups. 36.6% of patients were low, and 63.4% of patients were high. (A) Overall and (B) cancer‐specific survival were determined using standard Kaplan Meier methods. Patients with high 25D_3_ had improved survival (*P* = 0.007, 0.014, respectively). Comparisons were made using the log‐rank test

Having determined that VitD sufficient mice have an improved response to cisplatin treatment, we next investigated the effects of serum 25D_3_ levels on patients’ response to cisplatin. We analyzed serum 25D_3_ levels in a cohort of BCa patients who had been treated with cisplatin‐based chemotherapy. Out of 71 patients, 26 (36.6%) had low (deficient) 25D_3_ (<20 ng mL^−1^) and 45 (63.4%) had high (sufficient or greater) 25D_3_ (≥20 ng mL^−1^).^8^ Significant associations were found with overall (*P* = 0.007) and cancer specific survival (*P* = 0.014) where patients with high 25D_3_ had improved outcomes (Figure 3A‐B). This data confirms that VitD deficiency impairs patients’ response to cisplatin, and provides rational for serum 25D_3_ level to be used as a biomarker for cisplatin response.

### 3.5 1,25D_3_ increases the effect of cisplatin in BCa cell lines

**Figure 4:**
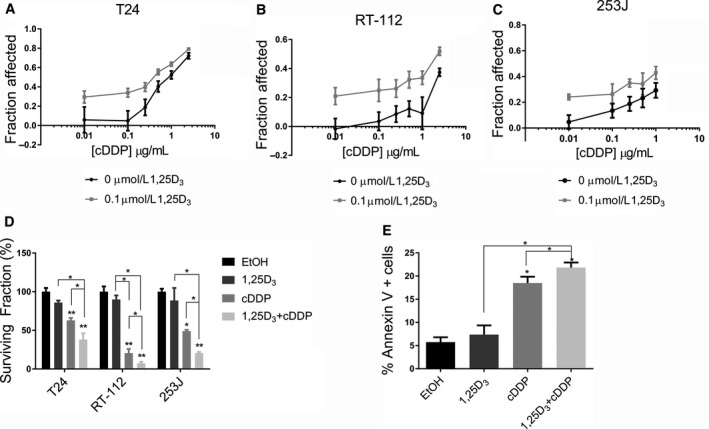
1,25D_3_ increases the response to cisplatin in vitro. (A) T24, (B) RT‐112, and (C) 253J cells were treated in triplicate with increasing concentrations of 1,25D_3_ (10 nmol L^−1^‐1 μmol L^−1^) for 24 hours followed by increasing concentrations of cDDP (0.01‐1 μg mL^−1^) for 48 hours and analyzed by MTT assay. Shown is the Fraction Affected (FA = 1‐OD treated cells/OD control cells) after treatment with 0 or 100 nmol L^−1^ of 1,25D_3_ followed by cDDP (n = 3). Pretreatment with 1,25D_3_ increased the FA compared with cDDP alone. (D) Clonogenic assays were performed in T24, RT‐112, and 253J cells treated with EtOH, 1,25D_3_ (100 nmol L^−1^), cDDP (0.1 μg mL^−1^), or the combination (n = 3). The surviving fraction decreased after the combination treatment compared with either monotherapy. (E) T24 cells were treated with EtOH, 100 nmol L^−1^ of 1,25D_3_, 0.1 μg mL^−1^ of cisplatin, or the combination and dual stained with Annexin V and 7‐AAD and analyzed by flow cytometry. The combination treatment increased the percentage of Annexin V+ cells. (n = 3, ANOVA, post‐hoc Bonferonni comparison, *P < 0.05, **P < 0.01)

To determine if 1,25D_3_ pretreatment can increase the effect of cisplatin in vitro and whether it is dependent on p53 status, T24 (mutant p53), RT‐112 (mutant p53) and 253J (wild type p53) BCa cells were treated with ethanol, 1,25D_3_ (10 nmol L^−1^‐1 μmol L^−1^), cisplatin (0.01‐1 μg mL^−1^), or 1,25D_3_ and cisplatin and analyzed by MTT. 1,25D_3_ pretreatment increased the fraction of cells affected (FA) by cisplatin, data shown with 100 nmol L^−1^ of 1,25D_3_ (Figure 4A‐C). 1,25D_3_ monotherapy had no effect (data not shown). Many combinations were found to be synergistic with a combination index (CI) < 1 by the Chou‐Talalay method.^28^ Remaining experiments use 100 nmol L^−1^ 1,25D_3_ and 0.1 μg mL^−1^ cisplatin which had a CI of 0.04 (T24), 0.35 (RT‐112), and 0.25 (253J). Next, clonogenic assays were performed to confirm the effects of 1,25D_3_ pretreatment on the response to cisplatin. Compared with cisplatin monotherapy, 1,25D_3_ and cisplatin combination decreased the surviving fraction from 62% to 38% (T24), from 21% to 7% (RT‐112), and from 49% to 20% (253J) (Figure 4D, *P* < 0.05). Representative images of colonies can be found in Figure S2. These studies confirm that pretreatment with 1,25D_3_ increases the response to cisplatin in vitro, independent of p53 mutation status.

### 3.6 1,25D_3_ and cisplatin combination therapy increases apoptosis

Our lab has shown previously that 1,25D_3_, cisplatin and gemcitabine combination can increase apoptosis. To verify this and investigate other mechanisms where VitD may affect cisplatin response we evaluated multiple aspects of cisplatin activity that may be altered by 1,25D_3_ pretreatment. Pretreatment with 1,25D_3_ did not significantly alter DNA‐platinum adduct formation (Figure S3A, *P* < 0.001) or repair (Figure S3B, *P* < 0.05), which has been linked to VitD.^29^ Nor did pretreatment with 1,25D_3_ affect cell cycle (Figure S3C). However, the percentage of apoptotic cells significantly increased from 5% in control treated cells to 17% with cisplatin monotherapy and to 22% with the combination (Figure 4E, *P* < 0.02). These results suggest that 1,25D_3_ pretreatment increases the anti‐tumor response to cisplatin by increasing apoptosis, consistent with prior data in a squamous cell carcinoma and pancreatic models.^26^, ^30^

### 3.7 1,25D_3_ pretreatment increases TAp73 expression

**Figure 5:**
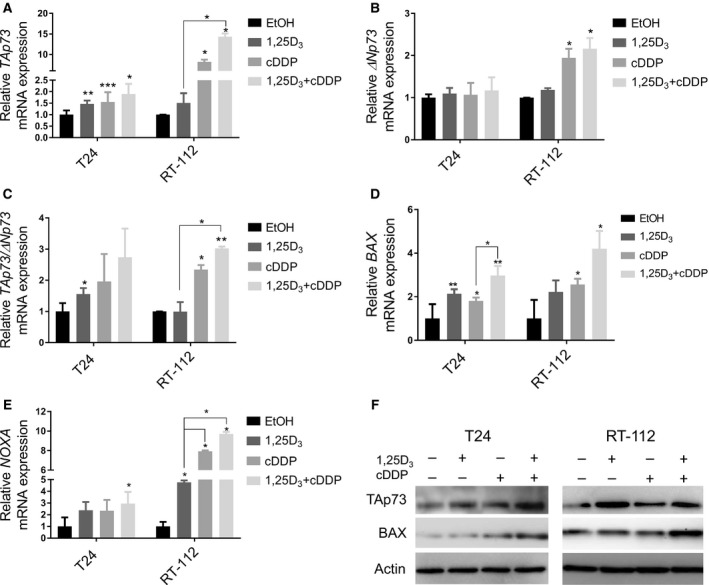
1,25D_3_ and cisplatin combination treatment increase TAp73 and targets. T24 and RT‐112 cells were treated with EtOH, 1,25D_3_ (100 nmol L^−1^), cDDP (0.1 μg mL^−1^), or the combination. (A) *TAp73* (B) *ΔNp73* (C) *TAp73/ΔNp73* (D) *BAX* and (E) *NOXA* mRNA expression was analyzed by qRT‐PCR. The combination treatment increased *TAp73*,*TAp73/ΔNp73, BAX, and NOXA* to the greatest degree in both cell lines. (F) TAp73 and BAX protein expression were analyzed by Western blot. The combination treatment increased TAp73 and BAX protein expression compared with cisplatin monotherapy. (n = 3, ANOVA, post‐hoc Bonferonni comparison, *P < 0.05, **P < 0.01, ***P < 0.001)

Because the combination treatment decreased the surviving fraction in all 3 cell lines, we hypothesized that p73, not p53, may be important in the apoptotic response. Our lab has previously shown p73 increases after treatment with 1,25D_3_ and cisplatin however the effects on individual isoforms of p73 were not investigated. The effects of treatment on TAp73 and ΔNp73 in cell lines with mutant p53, T24 and RT‐112, were analyzed. *TAp73* mRNA levels significantly increase after each treatment, with the greatest increase after the combination treatment in T24 and RT‐112 cells (Figure 5A, *P < 0.05, **P < 0.01). *ΔNp73* mRNA levels do not significantly change after treatment in T24 cells and slightly increase in RT‐112 cells after cisplatin treatment, alone or in combination with 1,25D_3_ (Figure 5B, *P < 0.05). The ratio of *TAp73/ΔNp73* increases approximately 3‐fold after the combination treatment in both cell lines (Figure 5C, *P < 0.05, **P < 0.01). These results suggest that the increase in apoptosis seen after 1,25D_3_ pretreatment may be in part due to the increased transcription of *TAp73* over *ΔNp73*. mRNA levels of transcriptional targets of TAp73, BAX and NOXA, and protein levels of BAX were found to increase after the combination treatment (Figure 5D‐F, *P < 0.05, **P < 0.01). TAp73 protein levels increase after the combination treatment in T24 and RT‐112 cells (Figure 5F). These observations confirm an increase in TAp73 expression and transcriptional activity by the combination treatment.

**Figure 6:**
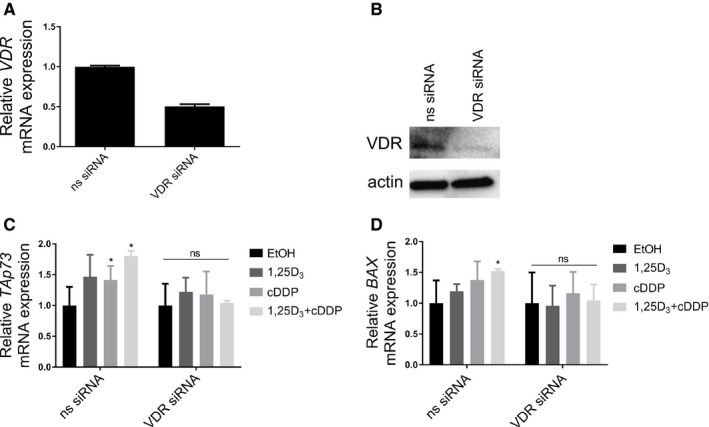
VDR is required for the induction of TAp73 and BAX after treatment with 1,25D_3_ and cisplatin. VDR was knocked down using siRNA in T24 cells. A decrease in both (A) mRNA and (B) protein expression was confirmed. Cells were treated with EtOH, 1,25D_3_ (100 nmol L^−1^), cDDP (0.1 μg mL^−1^), or the combination. qRT‐PCR was used to quantify transcriptional increases of (C) *TAp73* and (D) *BAX* after treatment. Induction was abrogated in VDR siRNA transfected cells

Finally, we tested the requirement for VDR in mediating the transcriptional increase in *TAp73* and *BAX* after treatment in T24 cells. VDR knockdown using siRNA (Figure 6A‐mRNA expression, B‐protein expression) prevented the induction of *TAp73* and *BAX* mRNA by either monotherapy or the combination (Figure 6C‐D, *P* < 0.05, normalized to ns‐shRNA EtOH treated (no decrease in TAp73 mRNA expression)). These data confirm that the combination treatment increases TAp73 expression and transcriptional activity, and that this induction requires VDR.

### 3.8 1,25D_3_ and cisplatin treatment requires TAp73

**Figure 7:**
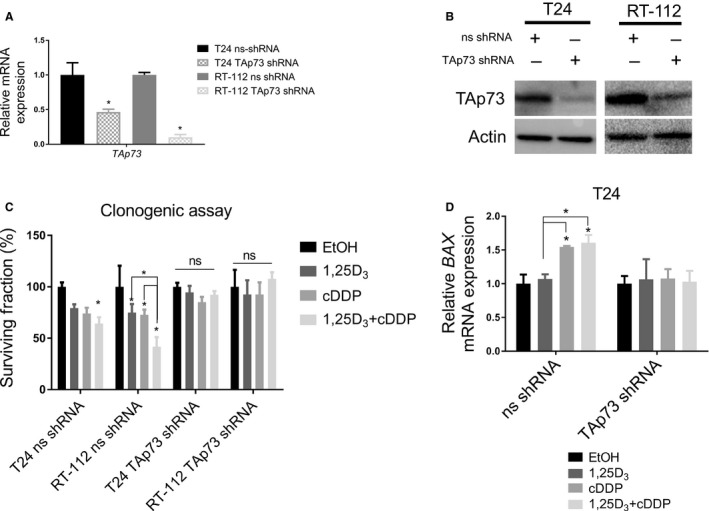
TAp73 is required for the effects of 1,25D_3_ and cisplatin combination treatment. TAp73 was knocked down in T24 and RT‐112 cells using shRNA. A decrease in both (A) mRNA and (B) protein was confirmed. Cells were treated with EtOH, 1,25D_3_ (100 nmol L^−1^), cDDP (0.1 μg mL^−1^), or the combination and clonogenic assays were performed. (C) The decrease in surviving fraction after 1,25D_3_, cisplatin, or the combination treatment was abrogated in TAp73 shRNA transduced cells. (D) BAX induction after treatment was confirmed in the ns shRNA tranduced cells, but not in TAp73 shRNA transduced cells. (n = 3, ANOVA, post‐hoc Bonferonni comparison, *P < 0.05)

Next, the requirement of TAp73 for the response to the combination treatment was determined. Two different shRNA constructs targeting TAp73 were transduced into T24 and RT‐112 cells and stable cell lines were generated (data shown with one per cell line, similar results in each). TAp73 shRNA transduction reduced mRNA and protein levels of TAp73 (Figure 7A‐B). The contribution of TAp73 in the clonogenic capacity was then examined by clonogenic assays. The non‐silencing control cells followed the pattern of the parental T24 and RT‐112 cell lines with the greatest reduction in surviving fraction found after the combination treatment (Figure 7C, *P* < 0.05). In TAp73 shRNA cells, all effects of treatment were abrogated. Surviving fractions ranged from 86%‐96% in T24 cells and 92%‐107% in RT‐112 cells compared with ethanol treated cells (Figure 7C, *P* < 0.05). Representative images from each cell line and treatment group are presented in Figure S4A. The transcriptional induction of *BAX* after the combination treatment was also abrogated after TAp73 knockdown, confirming that TAp73 contributes to the transcriptional regulation of BAX (Figure 7D, TAp73 shRNA normalized to EtOH, *P* < 0.05). These findings indicate that TAp73 is required for the reduction in surviving fraction by the combination treatment.

### 3.9 1,25D_3_ and cisplatin combination treatment increases the vitamin D_3_ response

In order to determine how combination treatment affects the VitD response, T24 and RT‐112 cells were treated with ethanol, 1,25D_3_, cisplatin, or 1,25D_3_ and cisplatin. Treatment with either 1,25D_3_ or cisplatin monotherapy increased VDR mRNA to a similar degree in both T24 and RT‐112 cells. The combination treatment significantly increased *VDR* mRNA expression compared with either monotherapy (Figure S5A‐B, *P < 0.05, **P < 0.01). VDR protein levels increase with 1,25D_3_ and further with 1,25D_3_ followed by cisplatin (Figure 7A). To determine whether increased VDR leads to a functional response, we analyzed two VDR target genes, *CYP24A1* and *cathelicidin antimicrobial peptide* (*CAMP*). Consistent with the effect on VDR expression, 1,25D_3_ alone increased mRNA expression of *CYP24A1* and *CAMP* and the combination resulted in a greater increase in expression (Figure S5C‐F).

### 3.10 TAp73 is required for the induction of VDR and CYP24A1

**Figure 8:**
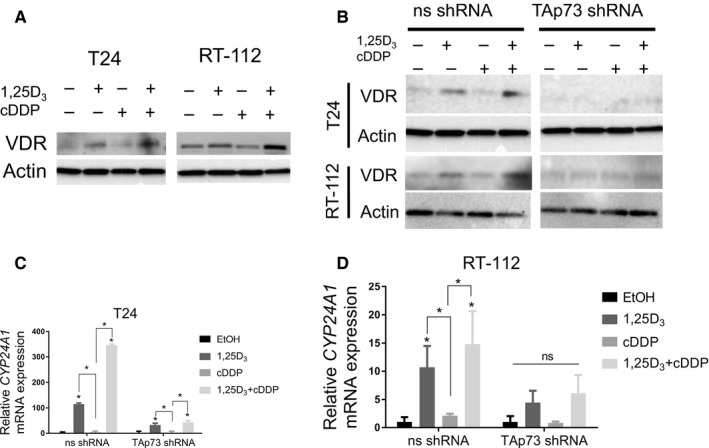
The vitamin D_3_ response requires TAp73. (A) T24 and RT‐112 cells were treated with EtOH, 1,25D_3_ (100 nmol L^−1^), cDDP (0.1 μg mL^−1^), or the combination. VDR protein expression was analyzed by Western blot analysis. VDR increased after 1,25D_3_ monotherapy, and further increased after the combination treatment. (B‐D) TAp73 shRNA transfected cells were treated with EtOH, 1,25D_3_ (100 nmol L^−1^), cDDP (0.1 μg mL^−1^), or the combination. Protein and mRNA expression were analyzed by western blot and qRT‐PCR. (B) 1,25D_3_ treatment increased VDR protein in ns shRNA transfected cells, but not TAp73 shRNA transfected cells. *CYP24A1* mRNA expression increased in (C) T24 and (D) RT‐112 ns shRNA transfected cells after 1,25D_3_ monotherapy or combination treatment. Induction was abrogated in TAp73 shRNA transfected cells. (n = 3, ANOVA, post‐hoc Bonferonni comparison, *P < 0.05)

Having discovered that TAp73 shRNA reduced the effects of 1,25D_3_ monotherapy on surviving fraction (Figure 6E), we next wanted to determine if TAp73 was required for VitD signaling. We treated T24 and RT‐112 cells transduced with TAp73 shRNA as described and analyzed the expression of VDR and *CYP24A1*. As expected, ns shRNA cells had a significant increase in *CYP24A1* mRNA expression and VDR protein expression after 1,25D_3_ and an even greater increase after the combination (Figure 8B‐D, *P* < 0.05). In contrast, in cells transduced with TAp73 shRNA, 1,25D_3_ alone or in combination with cisplatin was no longer able to induce VDR protein and *CYP24A1* mRNA levels to the same magnitude (Figure 8B‐D, *P* < 0.05). This result suggests that TAp73 protein is in part required for VitD signaling and may regulate VDR expression.

## 4 DISCUSSION

Treatment with cisplatin in advanced BCa patients has a limited response (5%‐10% improved survival over cystectomy).^31^, ^32^ Approximately 30%‐50% of BCa patients treated with cisplatin‐based chemotherapy will not benefit from therapy and 50% of patients treated with cystectomy develop metastatic disease within 2 years.^3^, ^4^, ^5^, ^31^, ^33^ New approaches to increase the efficacy of cisplatin are necessary to improve patient outcomes. We determined that VitD deficiency impairs the response to cisplatin, and that supplementation, either through dietary intervention or treatment with 1,25D_3_, can increase the response to cisplatin. It stands to reason that these results could be expanded to other tumor types, specifically those where incidence has been related to vitamin D deficiency such as prostate cancer.

**Figure 9:**
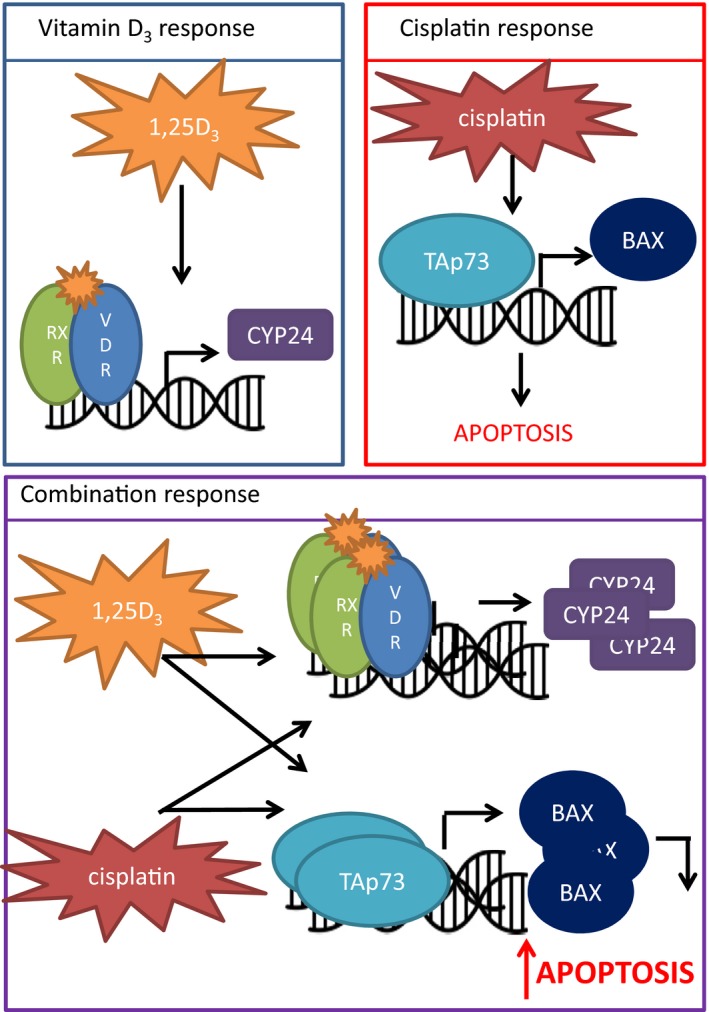
Diagram of mechanism of action: 1,25D_3_ monotherapy activates the transcription factor VDR, which heterodimerizes with retinoid‐X receptor (RXR) and transcribes target genes such as *CYP24A1*. Cisplatin monotherapy activates the transcription factor TAp73 which transcribes pro‐apoptotic target genes such as *BAX*. When we treat with the combination of 1,25D_3_ and cisplatin due to signaling crosstalk there is a greater increase in both VDR and TAp73 expression and as a result an increase in transcription regulation of *CYP24A1* and *BAX*, and ultimately the induction of apoptosis

The mechanism of action of 1,25D_3_ and cisplatin response is through VDR and TAp73 signaling crosstalk to increase the apoptotic response to cisplatin (Figure 9). We identified TAp73 as a key player in the response to 1,25D_3_ and cisplatin. This is consistent in the literature where p53 status is not linked to cisplatin response.^14^, ^23^ Pretreatment with 1,25D_3_ increases the ratio of TAp73/ΔNp73 mRNA, previously determined to be important in the response to chemotherapy.^20^, ^21^, ^22^ The expression of the transcriptional targets of TAp73, BAX and NOXA, increases with the combination treatment, in a VDR dependent manner. In addition, treatment with 1,25D_3_ and cisplatin appears to increase VitD response through VDR induction. The combination therapy had a greater effect on the expression of VDR and its targets, *CYP24A1* and *CAMP*, than 1,25D_3_ alone. It appears that in the presence of ligand, cisplatin can induce VDR protein expression, in a TAp73 dependent manner. This pathway crosstalk creates a positive feedback loop that results in an enhanced apoptotic response.

In the literature, there is evidence of crosstalk between VDR and p53 or p73.^34^, ^35^, ^36^ Our studies have identified VDR and TAp73 crosstalk in BCa cells as a result of treatment with 1,25D_3_ and cisplatin. An important limitation to our studies is the lack of mechanistic data in wild type p53 cell lines. Typically, cell lines with wild type p53 express very little p73, making changes in expression nearly impossible to detect. Due to high structural similarity between p53 and TAp73 functional domains, we hypothesize that crosstalk between VDR and p53 is possible and could explain the response to 1,25D_3_ and cisplatin in cell lines with wild type p53. Both VDR and TAp73 have been shown to physically interact with mutant p53 and alter normal function.^37^, ^38^ We hypothesize that our treatments may overcome this repression and restore VDR and TAp73 transcriptional regulation.

In summary, this study suggests that VitD plays a role in the response to cisplatin in BCa. VitD sufficiency, through dietary intervention or treatment with 1,25D_3_, can increase the therapeutic response to cisplatin. Patients who received cisplatin treatment with high 25D_3_ levels had improved survival. The mechanism of action of 1,25D_3_ and cisplatin anti‐tumor response is through TAp73 and VDR signaling crosstalk to potentiate apoptosis. Lastly, VitD deficiency may have potential use as biomarkers for response to cisplatin. Our data suggests that patients should be screened for their 25D_3_ levels prior to treatment and given intervention if they are found to be VitD deficient.

## CONFLICT OF INTEREST

None.
