# Supplementary figures and images for: Vitamin D3 enhances the response to cisplatin in bladder cancer through VDR and TAp73 signaling crosstalk
Source: Cancer Med. 2019 Apr 10;8(5):2449–61. doi: 10.1002/cam4.2119 (PMC6537042; doi:10.1002/cam4.2119)

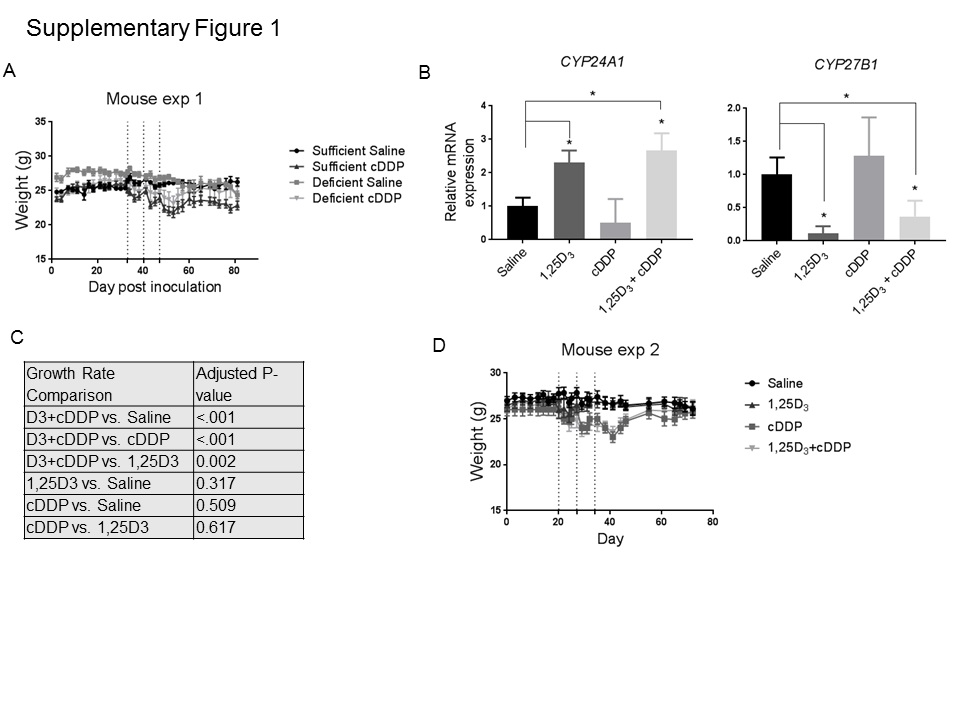

Supplement: Supplementary file 1 [file CAM4-8-2449-s001.TIF]

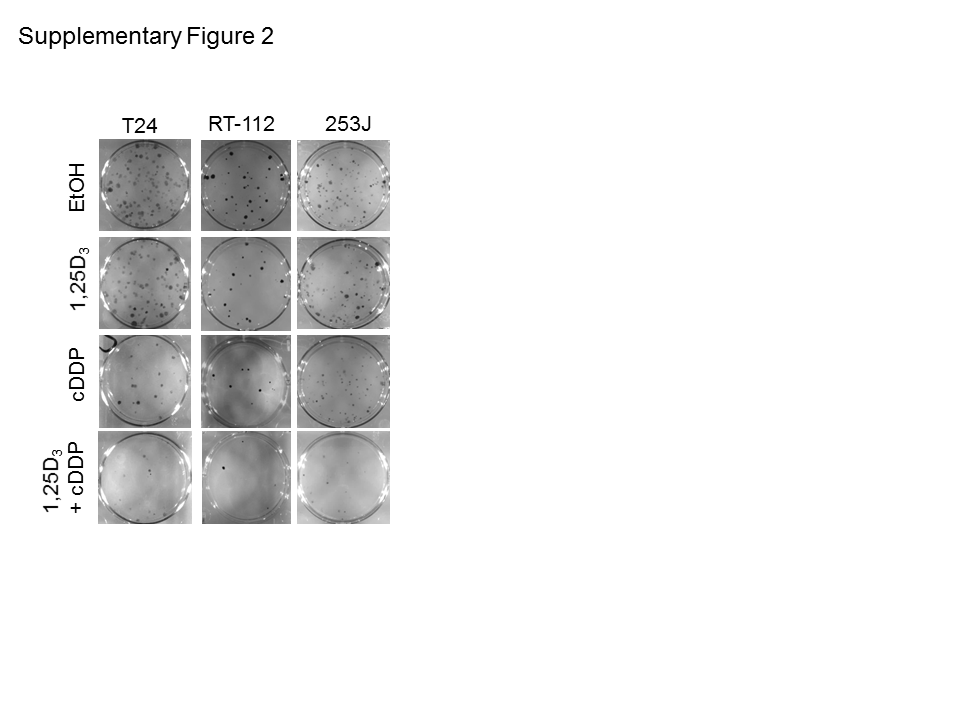

Supplement: Supplementary file 2 [file CAM4-8-2449-s002.TIF]

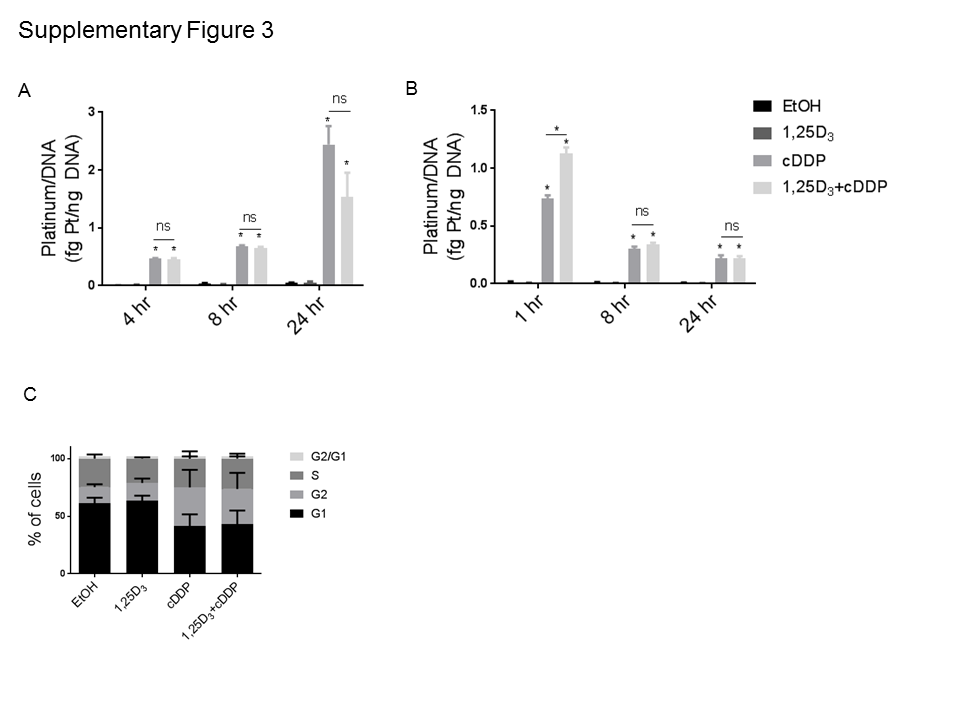

Supplement: Supplementary file 3 [file CAM4-8-2449-s003.TIF]

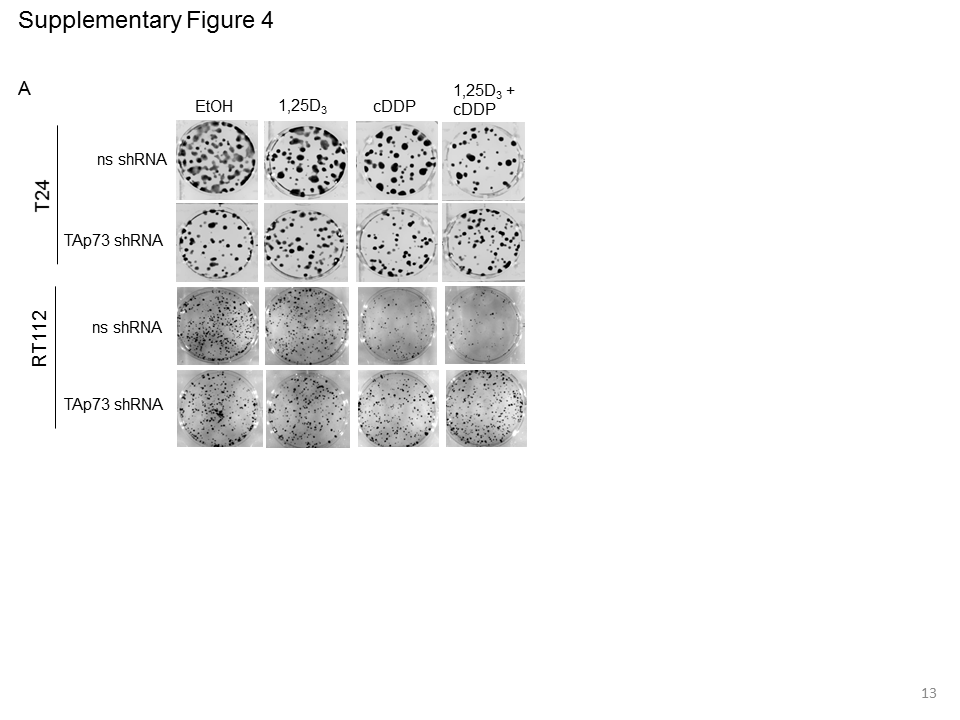

Supplement: Supplementary file 4 [file CAM4-8-2449-s004.TIF]

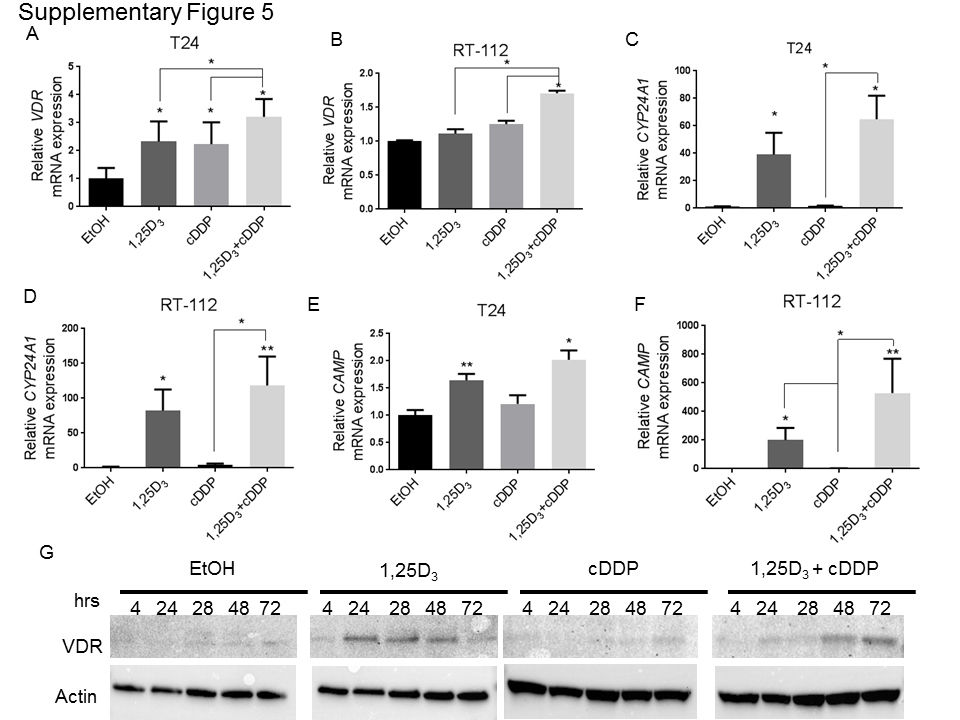

Supplement: Supplementary file 5 [file CAM4-8-2449-s005.TIF]

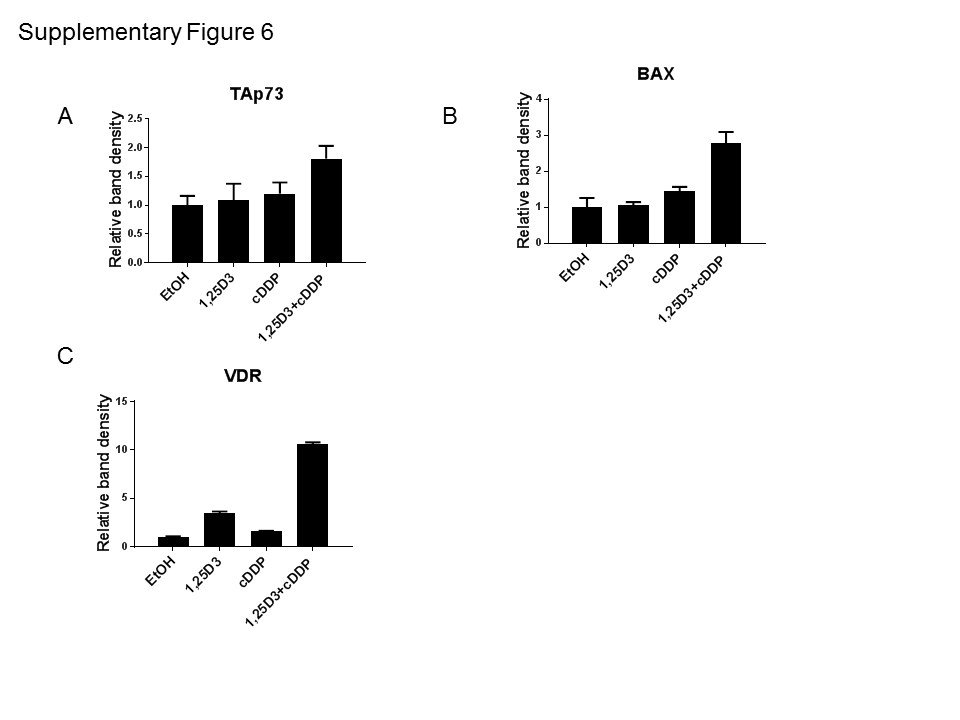

Supplement: Supplementary file 6 [file CAM4-8-2449-s006.tif]

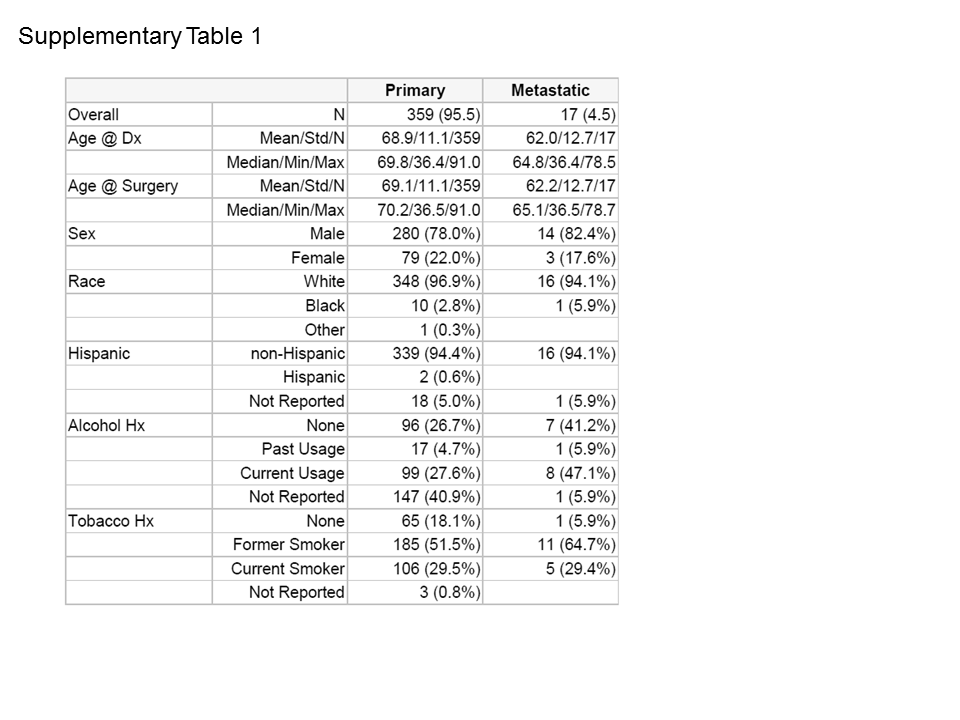

Supplement: Supplementary file 7 [file CAM4-8-2449-s007.TIF]

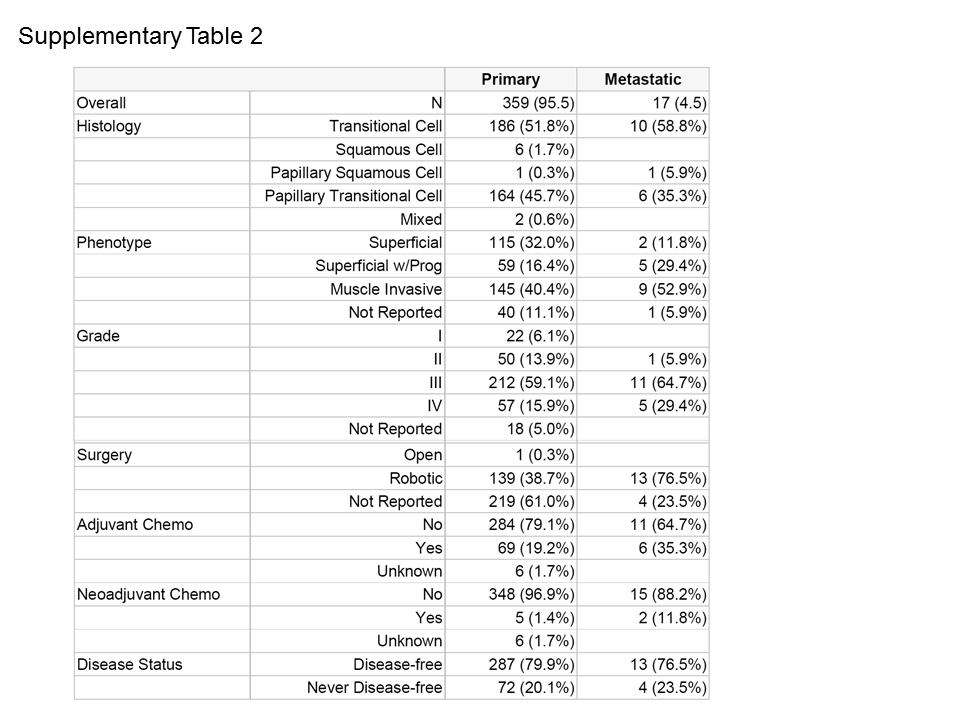

Supplement: Supplementary file 8 [file CAM4-8-2449-s008.TIF]

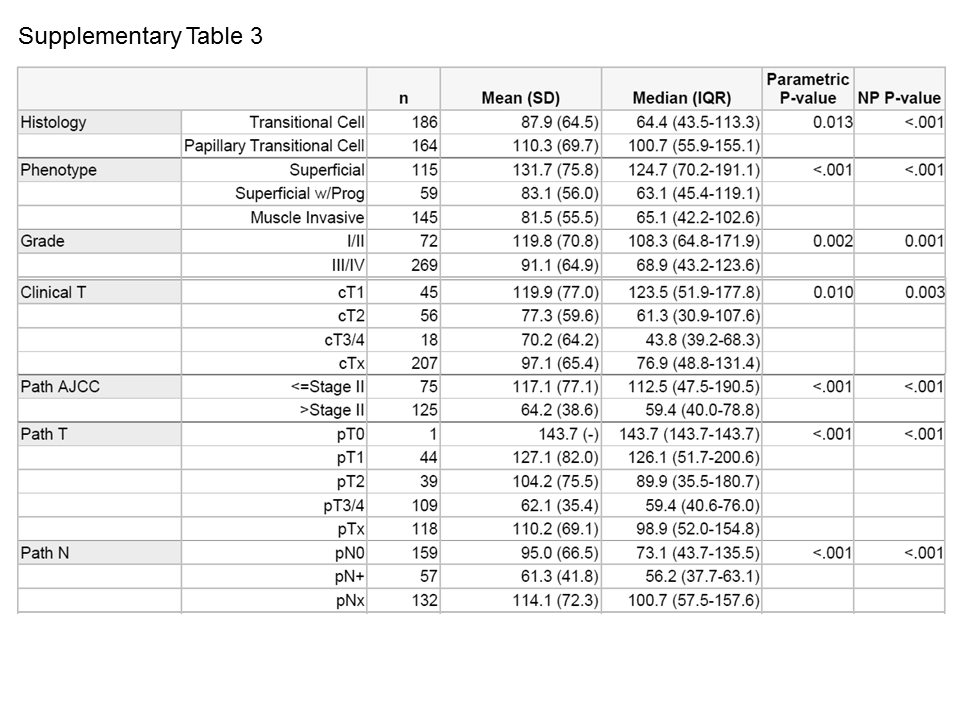

Supplement: Supplementary file 9 [file CAM4-8-2449-s009.TIF]
