# Supplementary material for: Vitamin D3 enhances the response to cisplatin in bladder cancer through VDR and TAp73 signaling crosstalk
Source: Cancer Med. 2019 Apr 10;8(5):2449–61. doi: 10.1002/cam4.2119 (PMC6537042; doi:10.1002/cam4.2119)
Supplement: Supplementary file 10 [file CAM4-8-2449-s010.docx]

**Supplementary Methods**

**Materials**

1,25D_3_ was a generous gift from Hoffmann-LaRoche (Nutley, NJ). Cisplatin (cDDP) was obtained from the Roswell Park Cancer Institute pharmacy (Buffalo, NY). Anti-p73 (A300-126A) was from Bethyl Laboratories (Montgomery, TX). Anti-VDR (D-6) and anti-BAX (N-20) were from Santa Cruz Biotechnology (Santa Cruz, CA). Anti-actin (CP-01) was from Calbiochem (San Diego, CA). Anti-PARP was from BD Pharmingen (San Jose, CA). Expression arrest pGIPZ lentiviral shRNAAmir-GFP human shRNA bacteria glycerol stocks (control, V2LHS_69894, V2LHS_181826, V3LHS_330454) were purchased from Thermo Scientific Open Biosystems. ON-TARGET plus Control pool (Cyclophilin B Human) and SMART pool (Human VDR) were purchased from Thermo Scientific Dharmacon®.

**Aperio analysis**

The Aperio platform does quantitative image analysis using algorithm macros to quantify immunohistochemistry (IHC) slides. The Nuclear algorithm detects the positive (DAB) nuclear staining for the individual tumor cells and quantifies their staining intensity. The analysis results provided the total number of detected cells, the percentage of cells per scoring class (0, 1+, 2+ and 3+) and the percentage of positive stained cells along with each samples average staining intensity of the positive nuclei as a score of 0, 1+, 2+ and 3+. The H-Score for each sample was calculated using the formula: (3 x percentage of strongly staining nuclei) + (2 x percentage of moderately staining nuclei) + (percentage of weakly staining nuclei), giving a range of 0 to 300.

**DNA/platinum adduct formation**

T24 cells were plated on day 0, treated with ethanol or 1,25D_3_ (100 nM) on day 1, and cDDP (0.1 μg/ml) on day 2. Cells were collected 4, 8, and 24 hrs after cisplatin was given and DNA was isolated using Qiagen midi kit (Qiagen, Gaithersburg, MD). DNA-platinum adducts were measured by Inductively Coupled Plasma-Mass Spectrometry (ICP-MS). Samples were digested with 40 µl of concentrated trace metal nitric acid (Fisher Scientific, Fairlawn, NJ) for 10 minutes in an 65ºC water bath. Samples were cooled to room temperature and 40 µl of 30% trace metal hydrogen peroxide (Fisher Scientific Optima grade) was added and incubated for an additional 10 minutes at 65ºC. Samples were diluted to a final volume of 400 µl with 2% nitric acid. Samples were analyzed by ICP-MS using the NexION 350D (Perkin Elmer, Shelton, CT) equipped with the microFAST system (Elemental Scientific Inc., Omaha, NE). Prior to analysis, nebulizer gas flow, torch alignment, and quadrupole ion deflector (QID) were optimized daily to pass the standard performance check. Iridium was used as the internal standard to control for instrument drift and was added inline (stock concentration 10 ppb) to each sample and standard. Quantitative analysis to determine platinum concentrations were set up using a 1 ppb platinum standard curve (0.1, 0.5, 1.0 ppb, Perkin Elmer). Platinum 195 and iridium 193 isotopes were measured in standard mode using peak hopping scan with 50 sweeps per reading and three replicates for each sample. Dwell times were 150 ms for platinum and 25 ms for iridium. DNA-platinum adducts were calculated by normalizing each platinum value to the DNA concentration.

**Cell cycle analysis**

Cells were treated with ethanol, 100 nM 1,25D3, 0.1 µg/ml cDDP, or 1,25D3 and cDDP. Cells were trypsinized and washed twice in PBS. Cells were then fixed in ice-cold 70% ethanol and vortexed. Samples were spun at 2000 rpm for 5 minutes and washed twice in PBS. Fixed cells were treated with 100 μl of 100 μg/ml ribonuclease for 5 minutes at room temperature. 400 μl of propidium iodide (50 μg/ml) was added to each sample. Samples were analyzed by flow cytometry using an LSRII and data was analyzed using Modfit software.

**Apoptosis**

T24 cells were plated in 6-well plates at a density of 100,000 cells per well and treated in triplicate as described. Cells were trypsinized and collected. PE Annexin V Apoptosis Detection Kit I protocol was used to determine the percentage of cells undergoing apoptosis. Briefly, cells were washed twice with cold PBS and resuspended in 1X Binding Buffer. 5 μl of PE Annexin V and 5 μl of 7-AAD were added to 100 μl of cell suspension and incubated for 15 minutes at room temperature in the dark. 400 μl of 1X Binding Buffer was added and samples were analyzed by flow cytometry using an LSRII. Data analysis was performed using FlowJo software.

**Primer sequences**

TAp73 (fw:5’-GCACCACGTTTGAGCACCTCT-3’ rev:5’-GCAGATTGAACTGGGCCATGA-3’), ΔN73 (fw:5’-AAGCGAAAATGCCAACAAAC-3’ rev:5’-CACCGACGTACAGCATGGTA-3’), BAX (fw:5’-CCCTTTTGCTTCAGGGTTTCA-3’ rev:5’-ACAGGGACATCAGTCGCTTC-3’), NOXA (fw:5’-GCTCCAAGTCGAGTGTGCTA-3’ rev:5’-GGAGTCCCCTCATGCAAGTT-3’), GAPDH (fw:5’-GTGGTCTCCTCTGACTTCAAC-3’ rev:5’-CCTGTTGCTGTAGCCAAATTC-3’), CYP24A1 (fw:5’-TTGGTCGCCGATTAGCAGAG-3’ rev:5’-ACCACCATCTGAGGCGTATT-3’), CAMP (fw:5’-GACAGTGACCCTCAACCAGG-3’ rev:5’-GGGCACACACTAGGACTCTG-3’), VDR (fw:5’-CAAAGCTGTGGGCTTTCGTTC-3’ rev:5’- TAGGCTTATGCCCCTGGTGT-3’), CYP27B1 (fw:5’-CAAGCAGCCGCGGGCTATGCTGG-3’ rev:5’-TGTCTGGGACACGGGAATTCC-3’).

**TAp73 shRNA sequence**

TGCTGTTGACAGTGAGCGAGGCCATGCCTGTTTACAAGAATAGTGAAGCCACAGATGTATTCTTGTAAACAGGCATGGCCCTGCCTACTGCCTCGGA

**VDR siRNA pool sequences**

GCAACCAAGACUACAAGUA, GCGCAUCAUUGCCAUACUG, CCAACACACUGCAGACGUA, GCAAUGAGAUCUCCUGACU

**Supplementary Figure Legends**

**Supplementary Figure 1:** (A) Twenty female nude mice on a VitD deficient (25 IU) or sufficient (1000 IU) diet were treated with 5 mg/kg of cDDP once a week for three weeks (n=5, Figure 2A-B). Mouse weights were monitored every other day. Mouse weights decreased slightly (5-7%) in VitD deficient and sufficient mice treated with cDDP. (B) Tumor growth rates (Figure 2C) were compared across treatment groups using an F-test. Post-hoc comparisons of growth rates was made using Holm-Berferroni adjusted F-test. VitD deficient mice treated with 1,25D_3_ and cisplatin had a significant reduction in tumor growth compared with either monotherapy. (C) Mouse weights from nude mice on a VitD deficient (25 IU) diet treated with saline, 1,25D_3_ (0.625 μg, MWF), cDDP (5 mg/kg, F), or the combination (n=10, Figure 2C). Mouse weights decreased slightly (7%) in mice treated with cDDP or the combination treatment.

**Supplementary Figure 2:** T24, RT-112, and 253J cells were treated with EtOH, 1,25D_3_ (100 nM), cDDP (0.1 μg/ml), or the combination. Clonogenic assays were performed in triplicate. Representative images of colonies after treatments are shown.

**Supplementary Figure 3:** T24 cells were treated with EtOH, 1,25D_3_ (100 nM), cDDP (0.1 μg/ml), or the combination. (A-B) DNA was isolated after cDDP treatment at different time points and DNA-platinum adducts were quantified using ICP-MS (A) 1,25D_3_ pretreatment does not affect DNA-platinum adduct accumulation or (B) DNA-platinum adduct repair. (C) Treated cells were stained with propidium iodide and analyzed for cell cycle phase by flow cytometry. cDDP treatment induced G2 phase accumulation. This was not altered by pretreatment with 1,25D_3_ (D-E) T24 and RT-112 cells were treated with EtOH, 1,25D_3_ (100 nM), cDDP (0.1 μg/ml, for 24, 48, and 72 hours), or the combination and caspase 3/7 activity was analyzed using a fluorescent dye. The combination treatment increased fluorescence. (ANOVA, post-hoc Bonferonni comparison, * p < 0.05, ** p < 0.01, *** p < 0.001)

**Supplementary Figure 4:** T24 and RT-112 cells were transfected with ns shRNA and TAp73 shRNA and treated with EtOH, 1,25D_3_ (100 nM), cDDP (0.1 μg/ml), or the combination. (A) mRNA expression of *TAp73* and *ΔNp73* decrease in TAp73 shRNA transfected T24 or RT-112 cells, determined by qRT-PCR. (B) Clonogenic assays were performed as described. Representative images of colonies after treatment in TAp73 shRNA transfected cells are shown here. (C) *BAX* mRNA expression was analyzed by qRT-PCR after treatment. Induction was abrogated in TAp73 shRNA transfected cells. (ANOVA, post-hoc Bonferonni comparison, * p < 0.05)

**Supplementary Figure 5:** T24 and RT-112 cells were treated with EtOH, 1,25D_3_ (100 nM), cDDP (0.1 μg/ml), or the combination. (A-B) *VDR*, (C-D) *CYP24A1,* and (E-F) *CAMP* mRNA expression was analyzed by qRT-PCR. Expression increased after 1,25D_3_ monotherapy and further increased after the combination treatment. (ANOVA, post-hoc Bonferonni comparison, * p < 0.05, ** p < 0.01)

**Supplementary Figure 6:** Western blot quantifications from three independent experiments of (A) TAp73, (B) BAX, and (C) VDR after treatment with EtOH, 1,25D_3_ (100 nM), cDDP (0.1 μg/ml), or the combination. Band densities were determined using ImageJ software and normalized to actin.

**Supplementary Table 1:** Patient demographics in the bladder cancer TMA

**Supplementary Table 2:** Patient treatment characteristics in the bladder cancer TMA

**Supplementary Table 3:** Patient clinical characteristics according to H-score
